# Environmental and Genetic Influences on Developmental Outcomes Across the Domains of Language, Cognition, Motor Function, and Social Behavior

**DOI:** 10.1111/ejn.70163

**Published:** 2025-06-17

**Authors:** Marta Schiavon, Birgitte K. Burton, Nicoline Hemager, Aja N. Greve, Katrine S. Spang, Ditte Ellersgaard, Kerstin Jessica Plessen, Jens Richardt M. Jepsen, Anne A. E. Thorup, Thomas Werge, Merete Nordentoft, Ron Nudel

**Affiliations:** ^1^ CORE – Copenhagen Research Center for Mental Health, Mental Health Center Copenhagen Copenhagen University Hospital Copenhagen Denmark; ^2^ iPSYCH, The Lundbeck Foundation Initiative for Integrative Psychiatric Research Aarhus Denmark; ^3^ Mental Health Center for Child and Adolescent Psychiatry – Research Unit Mental Health Services in the Capital Region of Denmark Copenhagen Denmark; ^4^ Department of Psychology University of Copenhagen Copenhagen Denmark; ^5^ Psychosis Research Unit Aarhus University Hospital – Psychiatry Aarhus Denmark; ^6^ Department of Clinical Medicine, Faculty of Health Aarhus University Aarhus Denmark; ^7^ Division of Child and Adolescent Psychiatry, Department of Psychiatry Hospital University Lausanne and Lausanne University Lausanne Switzerland; ^8^ Center for Neuropsychiatric Schizophrenia Research and Center for Clinical Intervention and Neuropsychiatric Schizophrenia Research Mental Health Services in the Capital Region of Denmark Glostrup Denmark; ^9^ Department of Clinical Medicine, Faculty of Health and Medical Sciences University of Copenhagen Copenhagen Denmark; ^10^ Institute of Biological Psychiatry Mental Health Center Sct. Hans, Mental Health Services Copenhagen Roskilde Denmark; ^11^ Copenhagen Research Center for Biological and Precision Psychiatry, Mental Health Center Copenhagen Copenhagen University Hospital Copenhagen Denmark

## Abstract

Linguistic, motor, cognitive, and social‐behavioral functions are fundamental facets of a child's neurodevelopment and are influenced by both genetic factors and environmental factors, such as the home environment, including the parents' mental health. However, the nature of these influences remains largely unknown. Using a genotyped cohort of 391 7‐year‐old children with comprehensive phenotype data on linguistic, motor, cognitive, and social‐behavioral performance as well as data on parental mental health and the home environment, we performed regression analyses for the individual neurodevelopmental domains and principal components (PCs) capturing the variance across all domains simultaneously, where these outcomes were regressed on a polygenic score for educational attainment (PGS for EA) as a proxy for genetic factors and the Home Observation for Measurement of the Environment (HOME) as a proxy for environmental factors. HOME was significantly associated with all domains; the PGS for EA was nominally significantly associated (*p* ≤ 0.05) with cognitive function only. In the principal component analysis, PC1 and PC2 captured 52.57% and 20.73% of the variance in our phenotypic data, respectively. HOME was significantly associated only with PC1, while the PGS for EA was significantly associated only with PC2. Significant differences between familial risk groups were observed for PC1. Our results suggest an important role for potentially modifiable environmental factors on child neurodevelopment across multiple domains. We identified two orthogonal dimensions capturing parts of phenotypic variance that were associated with either environmental or genetic factors, but not both, providing insight into the interplay between genes and the environment in neurodevelopment.

## Graphical abstract

We investigated the association between the home environment (HOME) and a polygenic score (PGS) for educational attainment (EA) with multiple developmental domains, both individually and, through principal component analysis (PCA), simultaneously. HOME was significantly associated with all developmental domains and with PC1, which captured most of the variance in the dataset, while the PGS for EA was significantly associated only with cognition and PC2.

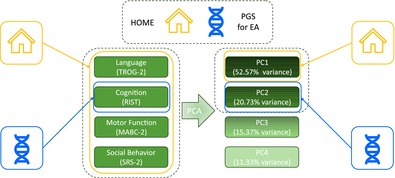

**Abbreviations**
ASD: Autism spectrum disorder
BP: Familial high risk of bipolar disorder
DCD: Developmental coordination disorder
EA: Educational attainment
FHR: Familial high risk (status)
HOME: Home Observation for Measurement of the Environment
HWE: Hardy–Weinberg equilibrium
MABC‐2: Movement Assessment Battery for Children, Second Edition
MAF: Minor allele frequency
PBC: Population‐based controls
PC: Principal component
PCA: Principal component analysis
PGS: Polygenic score
RIST: Reynolds Intellectual Screening Test
SD: Standard deviation
SRS‐2: Social Responsiveness Scale, Second Edition
SZ: Familial high risk of schizophrenia
TROG‐2: Test for Reception of Grammar, Second Edition

## 1 Introduction

Linguistic, motor, cognitive, and social‐behavioral functions are fundamental parts of a child's development, and they all develop dynamically and in concert with one another under the influence of both genetic and environmental factors (Blumberg et al. 2017). For example, while learning new motor skills may mediate the acquisition of perceptual information that results in new knowledge and facilitate social interaction (Adolph and Franchak 2017), cognitive skills are required to plan and guide such novel motor actions (Keen 2011) and are thus part of the same network of interconnected phenomena. Language and motor development are both correlated with cognition (Jin et al. 2023; Pereira et al. 2016) and with each other (Schiavon et al. 2024). Signs of the close relationship between language and motor skills emerge during infancy. In fact, learning to assume a sitting position and to walk independently is predictive of expressive vocabulary in infants between 16 and 28 months of age (Oudgenoeg‐Paz et al. 2012). Linguistic and motor ability are also crucial for inclusion in play activities with peers, and for the child's socialization (Eadie et al. 2018; Zwicker et al. 2013). More specifically, these two domains are believed to mediate each other's effect on children's social interactions: indeed, while children with poor motor skills are less likely to be invited to play and thus verbally interact with peers (Larkin and Summers 2004), a similar phenomenon affects children with language difficulties, who are also perceived as less attractive playmates and may therefore engage less frequently in active play (Fujiki et al. 1999; van der Niet et al. 2014). In addition to affecting one another, the previously mentioned domains are also influenced by a combination of genetic factors (as indicated by their heritabilities) and environmental factors. Heritability is defined as the ratio of the variance explained by genetic differences among individuals to the total trait variance in a population (Tenesa and Haley 2013). When the heritability is significantly above zero, the trait is considered heritable, but different human traits may vary greatly in their heritabilities. Intelligence, or cognitive ability, is generally thought to be heritable (Plomin and von Stumm 2018). Similarly, motor control and learning (Missitzi et al. 2013) and motor performance (Bouchard and Malina 1983) have also been found to be heritable. Social responsiveness is associated with common allelic variants and is also heritable (Nudel et al. 2022; Veddum et al. 2023). Lastly, in the case of language development, while shared environmental factors have a predominant role over genes in early language, this pattern is reversed in middle childhood, where genetic factors play a bigger role (Hayiou‐Thomas et al. 2012). Interestingly, educational attainment (EA), which is associated with all of these domains (Breslau et al. 2009; Durham et al. 2007; Hegelund et al. 2020; Kaufman et al. 2009; Keage et al. 2016; Lovden et al. 2020; Miles and Stipek 2006; Wang 2024), is also heritable (Lee et al. 2018; Shakeshaft et al. 2013). For some of the domains, specific genetic overlaps with EA exist as well, and genetic variants associated with both EA and language measures have been identified (Zhu et al. 2015); other studies have found genetic overlaps between EA and the morphology of brain regions related to language (Ge et al. 2019) and between EA and intelligence (Mitchell et al. 2022).

Although genetics undoubtedly plays an important role in linguistic, motor, cognitive, and social‐behavioral development, we must not underestimate the influence of the environmental context in which the child develops, and how early environmental factors may significantly contribute to the emergence of different phenotypes. For example, lower emotional and cognitive support assessed with selected items of the Home Observation for Measurement of the Environment, also known as the HOME inventory, mediated the negative effect of lower socioeconomic status on children's cognitive performance in socioeconomically disadvantaged families (Guo and Harris 2000). Similarly, children's cognitive abilities and early spoken language skills have been reported to be mediated by parental socioeconomic status and maternal higher education, respectively, with early spoken language skills showing a strong association with later academic performance during elementary school (Durham et al. 2007). Parent–child verbal and non‐verbal interactions are also crucial for the development of language and motor skills, respectively (Durham et al. 2007; Ku et al. 2023). By 36 months of age, children of parents who have attained a higher level of education and thus use a larger range of words in parent–child interactions, have a significantly larger vocabulary than children of less‐educated parents (Hart and Risley 1995). Exposure to different home environments and parenting styles also influences children's problem‐solving skills and patterns (Pettit et al. 1988) and, indirectly, their social competence with peers. Similarly, a positive home learning environment has been reported to be strongly correlated with children's social–emotional competence (Li et al. 2023). Overall, upper‐ and middle‐class children's better performance across multiple domains may also be ascribable to parenting practices which are more common in higher social strata, such as “concerted cultivation,” characterized by children's higher involvement in adult‐driven leisure activities and deliberately encouraging children to express themselves and interact with adults in a less subordinate fashion in comparison with what is observed in lower social classes. By contrast, working‐class families tend to value supporting children's natural growth through more independent play with peers and a clearer separation between children and adults (Lareau 2011).

Another important aspect of the child's home environment has to do with the parents' mental health. Children of parents affected by severe mental illness, and thus at higher risk of developing severe mental illness themselves, may show significant impairments in each of the previously mentioned domains (Burton et al. 2017; Christiani et al. 2019; Greve et al. 2024; Hemager et al. 2018). Although there is increasing evidence of at least some of these deficits representing an expression of the child's risk of developing severe mental illness (Poletti 2023), the interplay between genetic and environmental factors contributing to these early neurodevelopmental abnormalities and to poorer mental health outcomes has not been fully elucidated. Interestingly, the home environment is also associated with each of the four investigated domains (Ferreira et al. 2018; Guo and Harris 2000; Price et al. 2013; Ronfani et al. 2015). It need also be mentioned that, for some psychological traits and psychiatric disorders, gene–environment interactions exist, meaning that a genetic effect may in some way be dependent on the environmental context, or vice versa (Caspi and Moffitt 2006; Dick 2011).

In this study, our two main goals were (i) to use proxies for environmental factors and genetic factors which capture specific aspects of the child's home environment and the child's genetic propensity for EA, respectively, to assess the associations between these factors and linguistic, motor, cognitive, and social‐behavioral functions, both individually and as captured by principal components, i.e., orthogonal dimensions capturing the variance across children, and (ii) investigate whether and how these dimensions are associated with familial risk of severe mental illness. We leveraged data from a cohort of 391 children with familial risk of severe mental illness (schizophrenia spectrum disorders or bipolar disorder) or population‐based controls, with phenotype data across all four domains as well as genetic data. Using a polygenic score (PGS) for EA and the HOME inventory total score, our study assesses the influence of genetic and environmental factors on both the scores from the individual domains and the principal components computed in order to capture the variance across the children for all developmental domains simultaneously.

## 2 Methods

### 2.1 Participants

The children included in this study participated in The Danish High Risk and Resilience Study (VIA 7) (Thorup et al. 2015), a cohort consisting of 522 7‐year‐old children with at least one biological parent affected by a schizophrenia spectrum disorder (*N* = 202) or bipolar disorder (*N* = 120), and children of parents with neither disorder (population‐based controls [PBCs]) (*N* = 200), who underwent an extensive test battery which included neurocognitive, linguistic, motor, and social‐behavioral tests and questionnaires. The children and their families were identified using Danish national registries based on the presence or absence of a parental diagnosis (of a schizophrenia spectrum disorder or bipolar disorder), for the case and control subjects, respectively. The children included in this study are a subset of the cohort, comprising 391 unrelated children for whom we also had genetic data and who passed genetic quality control, which had been used in previous studies (Nudel et al. 2021; Nudel et al. 2020a). Population‐based controls were matched to the children from the group at familial high risk of schizophrenia on age, sex, urbanicity and geographical location—the last two could be seen as proxies for socioeconomic status—and children from the group at familial high risk of bipolar disorder did not significantly differ from those groups in terms of age and sex (Krantz et al. 2024; Thorup et al. 2015).

### 2.2 Phenotypes

The phenotypes included in this study for the domains of language, motor function, cognition, and social behavior are as follows: the standardized score from the second edition of the Test for Reception of Grammar (TROG‐2) (Bishop 2003), the standardized total score from the second edition of the Movement Assessment Battery for Children (MABC‐2) (Henderson et al. 2007), the standardized index score from the Reynolds Intellectual Screening Test (RIST) (Reynolds and Kamphaus 2003), and the T‐score from the Danish version of the second edition of the Social Responsiveness Scale (SRS‐2), completed by the child's teacher (Constantino et al. 2003). Descriptive statistics for the phenotype data can be found in Table 1. In addition to the phenotype scores themselves, we also computed principal components of the individual scores, as detailed below.

**TABLE 1:** Descriptive statistics for language, motor function, cognition, social behavior, and home environment for the 391 children included in the analysis.

	Minimum value	Maximum value	Mean	Median	Standard deviation	Number of missing values
Language (TROG‐2)	55	130	101.17	105	15.69	2
Motor function (MABC‐2)	1	19	8.17	8	3.31	2
Cognition (RIST)	56	127	103.85	105	10.41	1
Social behavior (SRS‐2)	37	106	49.08	46	10.53	56
HOME	31	58	47.12	48	5.35	3

### 2.3 Principal Component Analysis

We performed a principal component analysis (PCA) using the *prcomp* function (with centering and scaling) in R v4.2.2 (R Core Team 2014). PCA is a statistical method that identifies new orthogonal variables in the dataset (principal components [PCs]) which are linear combinations of the original variables such that the first PC, PC1, captures the highest amount of the variance in the data, followed by PC2, and so on. By definition, PCs are orthogonal to each other. Prior to the PCA, we performed an imputation of missing scores among the children using the *imputePCA* function of the missMDA package v1.19 (Josse and Husson 2016). The four phenotype scores were included as variables in the imputation. PCA biplots were generated with the packages ggplot2 v3.5.0 (Wickham 2016), ggfortify v0.4.16 (Tang et al. 2016), and gridExtra v2.3 (Auguie 2017). We retained the first two principal components for downstream analyses, as they captured more than 70% of the cumulative variance, which is an acceptable threshold for PCA (Jolliffe 2002). However, we additionally employed multiple formal methods to validate this choice using the *VSS* function of the R psych package v2.2.9 (Revelle 2022) on the phenotype matrix after the imputation (we note, however, that some of these methods are more appropriate in factor analysis than in PCA). The consensus from all the methods was that none retained PC3 or PC4. See note in the Supporting Information for the results of this analysis, and Table S1 for further information about the PCs. Note that the *prcomp* function in R returns the loadings (or rotations) of the principal components in the sense of coefficients for the (standardized) original variables, which are used to obtain the PC scores. This is the sense in which we use the term “loading” in this study, which may be different from how it is used in other contexts.

### 2.4 Genetic Data and Quality Control

DNA samples were genotyped on the Illumina PsychChip v1‐1_15073391_C. The full quality control steps for the genetic dataset were described in detail elsewhere (Nudel et al. 2020b,  2022). Briefly, markers and samples with > 5% missingness were removed; markers with a Gentrain score < 0.3 were removed; samples with discordant sex information were removed; markers and samples with > 1% Mendelian errors were removed, and remaining genotypes with errors were set to missing; samples with extreme heterozygosity (threshold of ±3 standard deviations [SDs] from the mean across VIA 7 samples) were removed; samples of divergent ancestry, identified through a PCA with continental HapMap populations and VIA 7 samples (threshold: 2 SD above or below the mean of PC1 and PC2 across the VIA samples), were also removed; samples showing cryptic relatedness (Pi‐hat threshold of 0.185) were removed, as were samples not genetically related to their relatives as per pedigree information; lastly, markers with Hardy–Weinberg equilibrium (HWE) *p* value < 10^−6^ or with minor allele frequency (MAF) < 1% in founders were removed. Only autosomal markers were used in downstream analyses. The final dataset had 299,604 markers and 1094 individuals (parents and children). Imputation of further genetic markers was conducted, as described previously (Jefsen et al. 2022). Briefly, after processing of genotype data with the QC script “HRC or 1000G Imputation preparation and checking” v.4.2.9 (http://www.well.ox.ac.uk/~wrayner/tools) and the HRC reference v1.1 for genome build GRCh37, phasing and imputation were conducted on the Michigan Imputation Server (Das et al. 2016) with the following parameters: Reference Panel: HRC r1.1 2016; Phasing: Eagle V2.4 (phased output); Population: EUR; Algorithm: Genotype Imputation (Minimac4) 1.5.7; Mode: QC and imputation. Following the imputation, hard call “best guess” genotypes were kept for genotypes with probabilities of at least 0.9. Imputed markers were then removed if: they had rsq < 0.3; they were duplicates of other markers; they were multiallelic; they were indels; they had Mendelian error rates exceeding 1% (genotypes with Mendelian errors below this threshold were set to missing); they had MAF < 1% in founders; they had a missingness rate > 5%; they had a HWE *p* value < 10^−6^. Marker IDs were changed to rsIDs based on the HRC reference, where possible. The final dataset had 5,828,122 markers. Following the imputation polygenic scores (PGS) for educational attainment (EA) were generated with PRSice v.2.2.3 (Choi and O'Reilly 2019) with the following parameters: clumping window of 250 kb and *r* ^2^ of 0.1; scoring method: score sum; otherwise, the default parameters were used. The *p* value threshold used was pT = 1. The summary statistics for EA were obtained from a large‐scale genetic study of EA, where the increase in *R* ^2^ for predicting EA in a separate cohort when using a PGS for EA was 9.4%, employing a similar method and the same *p* value threshold as in our study (Lee et al. 2018). The scores were standardized across the 391 unrelated children included in this study, such that the units of the PGS are in units of standard deviations from the mean PGS of the sample, with the *scale* function in R (with the default parameters). Table S2 includes the means and standard deviations for the PGS for EA across the familial risk groups.

### 2.5 Assessment of the Home Environment

The children's home environment was assessed using the HOME inventory (Bradley et al. 2003), a semi‐structured interview lasting approximately one hour, performed in the child's home with both the child and their primary caregiver present. The HOME inventory comprises several sections which capture the cognitive, physical, and verbal stimuli the child is exposed to in the home, and the level of parental emotional support they receive (Bradley et al. 2003). In this study, we used the Middle Childhood HOME inventory (hereafter: HOME), designed for the assessment of the home environment of children between ages 6 and 10. HOME comprises 59 items which are scored based either on the assessor's observations of the environment and/or child‐caregiver interactions, or the caregiver's answers to the assessor's interview questions. Each item investigates the presence of a specific behavior, material object, or condition. A higher HOME score indicates a higher appropriateness of the child's domestic environment. Further details on the collection of HOME data in the VIA 7 study are described elsewhere (Gantriis et al. 2019). Table S2 includes the means and standard deviations for the HOME score across the familial risk groups.

### 2.6 Statistical Analyses

Statistical analyses were performed in R. We performed linear regressions using the *lm* function, whereby either the phenotype scores or the principal components (PCs) were regressed on the HOME score, the PGS for EA and covariates for sex and the child's familial high‐risk status for mental illness. We also repeated the regressions while removing either the HOME score or the PGS for EA, where appropriate, to isolate the contribution of one of the two variables to the model using the R^2^s of the nested models. Confidence intervals were computed with the *confint* function. Analyses of variance to test differences between familial high‐risk groups or males and females were performed with the *aov* function. Lastly, to test for whether adding a term for an interaction effect between the HOME score and the PGS for EA improved the model, an F test using the *anova* function (*test=“F”*) with two nested models (one model with predictors for HOME, PGS for EA and the covariates, and one with an added term for the interaction) was performed for each phenotype and for PC1 and PC2.

## 3 Results

### 3.1 Linear Regressions of the Individual Traits on the HOME Score and the PGS for EA

We performed linear regressions to assess the impact of the HOME score and the PGS for EA on each developmental domain of interest. It should be noted that, in the case of SRS‐2, a higher score indicates poorer social responsiveness, whereas in the assessments used for the three other domains i.e., RIST, MABC‐2, and TROG‐2, a higher score is interpreted as better cognitive, motor, or linguistic performance, respectively. With regards to the predictors, a higher PGS indicates higher genetic predisposition to educational attainment, and a higher HOME score indicates higher quantity and quality of stimuli and support in the child's domestic environment. Cognition, indicated by the children's RIST index, was the only domain for which PGS for EA showed a nominally significant association, whereby a higher genetic predisposition to educational attainment was associated with a higher intelligence index. HOME was significantly positively associated with the RIST index, MABC‐2 total score, and TROG‐2 score, and negatively associated with the SRS‐2 score, meaning that a higher HOME score was associated with better performance across all domains individually (Table 2). Adding an interaction term between HOME and PGS for EA to the regression did not significantly improve the model for any phenotype: TROG‐2 (*F*(1, 379) = 0.2916, *p* = 0.5895); MABC‐2 (*F*(1, 379) = 0.5929, *p* = 0.4418); RIST (*F*(1, 380) = 0.1655, *p* = 0.6844); SRS‐2 (*F*(1, 326) = 1.1498, *p* = 0.2844).

**TABLE 2:** Results of the linear regression analyses of the traits of interest (language, motor function, cognition, and social behavior) and principal components on standardized polygenic scores (PGS) for educational attainment (EA) and HOME, with covariates for sex and familial high‐risk status.

Outcome	Coeff. for standardized PGS for EA [95% CI]	*p* value for coeff. for standardized PGS for EA	Coeff. for HOME [95% CI]	*p* value for coeff. for HOME	*R* ^2^ full model	*R* ^2^ for PGS for EA^a^	*R* ^2^ for HOME^a^
Language (TROG‐2)	0.696 [−0.859; 2.251]	0.379	0.648 [0.344;0.953]	3.48 × 10^−5^	0.096	0.002	0.041
Motor function (MABC‐2)	−0.154 [−0.477; 0.169]	0.349	0.140 [0.076;0.203]	1.87 × 10^−5^	0.122	0.002	0.043
Cognition (RIST)	1.301 [0.274;2.328]	0.013	0.484 [0.284;0.685]	2.94 × 10^−6^	0.104	0.015	0.055
Social behavior (SRS‐2)	−0.025 [−1.151;1.101]	0.965	−0.501 [−0.716;‐0.286]	6.79 × 10^−6^	0.123	0	0.057
PC1	−0.063 [−0.199;0.074]	0.367	−0.088 [−0.115;‐0.062]	2.07 × 10^−10^	0.188	0.002	0.091
PC2	0.114 [0.022; 0.207]	0.016	−0.005 [−0.024;0.013]	0.555	0.046	0.015	0.001

Abbreviations: CI, confidence interval; PC, principal component; PGS for EA, polygenic score for educational attainment.

^a^ The *R* ^2^ for specific predictors was calculated from the *R* ^2^ for the full model minus the *R* ^2^ for a model with all predictors except for the one in question.

### 3.2 Principal Component Analysis

**FIGURE 1:**
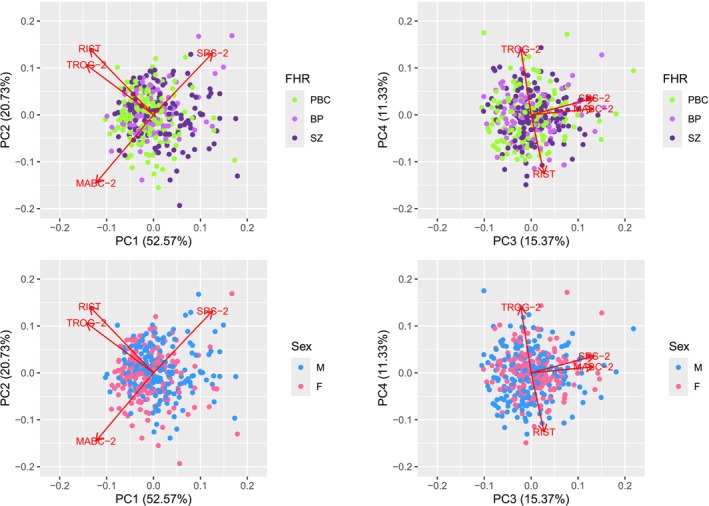
Biplots showing values of principal components 1–4 across groups stratified on sex or familial high‐risk status. The red arrows indicate the relationships between the principal components and the original variables (the scores across the four developmental domains). PC, principal component; M, male; F, female; FHR, familial high‐risk status; SZ, familial high risk of schizophrenia; BP, familial high risk of bipolar disorder; PBC, population‐based controls.

In the PCA, using scores for the four domains, four PCs were computed. PC1 and PC2 captured 52.57% and 20.73% of the variance in our data, respectively, amounting to 73.30% of the variance in the data. While this suggested that retaining these PCs for downstream analysis was appropriate, we additionally tested this using multiple common approaches (see Methods). In terms of the composition of the PCs, for PC1, TROG‐2 and RIST were the variables with the largest loadings in terms of absolute value. TROG‐2, MABC‐2 and RIST had high negative loadings, suggesting that PC1 represents a dimension where these three variables vary together. SRS‐2 had a positive loading for PC1. For PC2, MABC‐2 had a negative loading while all other variables had positive loadings. Of the other three variables, the variable with the largest positive loading in PC2 was RIST. It should be kept in mind that, while a higher score indicates a better performance in RIST, TROG‐2, and MABC‐2, the opposite is true for SRS‐2, i.e., a lower score indicates better social responsiveness, and the loading of SRS‐2 should be interpreted accordingly. Given that scaling and centering was employed prior to the PCA, the PC loadings indicate the contributions of the individual variables to the PCs; when multiplied by the square roots of the eingenvalues (Table S1), correlations between the PCs and the standardized original variables can be obtained. Figure 1 shows biplots of the PCs in relation to familial risk of mental illness and sex. Analyses of variance revealed significant group differences between familial high‐risk groups for PC1 (*F*(2, 388) = 9.776, *p* = 7.21 × 10^−5^), but not for PC2 (*F*(2, 388) = 1.673, *p* = 0.189), and significant group differences for both PC1 (*F*(1, 389) = 15.36, *p* = 0.0001) and PC2 (*F*(1, 389) = 8.54, *p* = 0.004) between males and females. For PC1, the mean in the schizophrenia group (mean = 0.39; standard deviation (SD) = 1.63) was higher than the mean in the bipolar disorder group (mean = −0.03; SD = 1.42), which, in turn, was higher than the mean in the population‐based control group (mean = −0.34; SD = 1.20). The means in males were higher than the means in females for both PC1 (males: mean = 0.26; SD = 1.47; females: mean = −0.31; SD = 1.37) and PC2 (males: mean = 0.12; SD = 0.88; females: mean = −0.15; SD = 0.93).

### 3.3 Linear Regressions of the Principal Components on the HOME Score and the PGS for EA as Proxies for Environmental and Genetic Predictors

We proceeded by performing linear regressions on PC1 and PC2 to see how these may be associated with HOME and the PGS for EA. HOME was significantly associated only with PC1, while the PGS was significantly associated only with PC2. Comparing the *R* ^2^ values across the regressions for the individual traits and the two PCs, we see that the predictors in the regression of PC1 explain the highest proportion of the variance (*R* ^2^ = 0.188), and that the independent variable that explains the most variance between HOME and the PGS for EA is HOME (*R* ^2^ = 0.091 for PC1). The results for all regressions are shown in Table 2. Adding an interaction term between HOME and PGS for EA to the regression did not significantly improve the model for PC1 (*F*(1, 381) = 0.882, *p* = 0.3482) or PC2 (*F*(1, 381) = 0.2325, *p* = 0.63).

## 4 Discussion

In our study of environmental and genetic influences on four neurodevelopmental domains, both separately and assessed simultaneously by PCA, we found that the child's HOME score was significantly associated with outcome measures across all four of the developmental domains of interest. In contrast, we only found a nominally significant association (i.e., not surviving Bonferroni correction for the regressions of the four scores) between genetic predisposition for educational attainment (PGS for EA) and cognitive function, yet no significant association with linguistic, motor, or social‐behavioral functions. With regards to the PCs, HOME was significantly associated only with PC1, while the PGS for EA was significantly associated only with PC2, suggesting that these two dimensions capture parts of phenotypic variance that are associated with either the home environment or genetic propensity for EA, but not both. Previous analyses on VIA 7 data have focused on group differences in single domains (including language, motor skills, social behavior, and cognition) and found that children of parents with schizophrenia show poorer performance in the four domains in comparison to PBC (Burton et al. 2017; Christiani et al. 2019; Greve et al. 2024; Hemager et al. 2018). Pertinent to this observation, it was found that the proportion of children living in an inappropriate home environment was significantly higher among children of parents with schizophrenia (Gantriis et al. 2019). Here, by employing PCA, in addition to looking at the individual domains, we identified new dimensions (the PCs) capturing variance in the data. The advantage offered by PCA in comparison with the investigation of single, separate neurodevelopmental domains lies in its ability to capture and visualize otherwise hidden patterns in the data, which may offer new insights into the multifaceted, yet concerted process of neurodevelopment. Furthermore, the PCs generated through PCA, in contrast to the original variables, are inherently uncorrelated. This design allowed us to identify orthogonal dimensions, which could reveal otherwise hidden associations with the predictors. Upon computing the PCs, we checked whether there were any differences across groups stratified based on familial risk of severe mental illness or sex with regards to these new dimensions. Additionally, in contrast to previous studies, we modeled the relationships between HOME and PGS for EA, on the one hand, and these four developmental domains (individually or within the PCA framework) on the other hand, using regression models. The results of the linear regressions for PC1 suggest that this dimension, capturing just over 50% of the variance across children, is associated with general development across the four domains of interest, and the directions of the loadings suggest that these domains may be influencing each other in the sense that children with high values for PC1 may show worse performance across all domains. Interestingly, the variance captured by PC2 unveiled a different pattern, where different domains influenced the composition of the PC in different directions, with MABC‐2 pulling in one direction and the other scores pulling in the other direction (keeping in mind that a higher SRS‐2 score indicates poorer social responsiveness). Analyses of variance revealed significant differences in PC1 across familial high‐risk groups, and significant differences in both PCs between males and females. Given the higher values of PC1 in children at familial high risk of schizophrenia, this suggests that these children may be at higher risk of general developmental deficits in multiple domains.

The linear regressions which investigated the effects of environmental factors (HOME) and genetics (PGS for EA) on PC1 and PC2 showed that HOME was significantly associated with PC1, and the PGS for EA was significantly associated with PC2. The significant negative association between HOME and PC1 indicates that higher stimulation and more appropriate child–caregiver interaction closely relate to better developmental outcomes across all domains simultaneously, as captured by this PC. Meanwhile, the significant positive association between the PGS for EA and PC2 indicates that genetic predisposition for EA is positively associated with a dimension with positive loadings for language and cognition. That in itself is not surprising; however, PC2 also had a positive loading for SRS‐2 and a negative loading for MABC‐2, which are suggestive of a higher probability of autism spectrum disorder (ASD), indicated by poorer social responsiveness, and worse motor function, respectively. In this context, it should be mentioned that a previous study has found a significant and positive genetic correlation between ASD and higher educational attainment (Hagenaars et al. 2016), which our result is in line with. While ASD and developmental motor disorders such as developmental coordination disorder (DCD) often present together and with similar motor and social‐behavioral difficulties, a past study has shown that children with DCD and suspected or confirmed ASD had significantly higher SRS‐2 scores than children with DCD alone (De Roubaix et al. 2024). This suggests the possibility of identifying a group of children with similar traits with PC2. Past studies have also sometimes revealed a negative association between gross motor skills and academic achievement or language (although, for fine motor skills, the association was always positive) (Wang 2024). The nature of these associations remains elusive, but we hypothesize that at least some children who perform poorly on motor tasks may be more inclined to spend more time on academic activities; a comprehensive study of the relationships between physical activity, sedentary time and academic performance found a strong association between higher sedentary time and higher academic performance (Maher et al. 2016). In the case of both PC1 and PC2, the PCs were associated with HOME and PGS for EA differently, as compared with the individual traits, revealing environmental and genetic factors which are likely to be affecting the neurodevelopmental process in its integrity. Interestingly, our analyses did not find significant evidence for an interaction effect between the PGS for EA and the HOME score for the individual domains or the PCs. In a previous study, we found nominally significant evidence for the effect of an interaction between motor impairment and familial risk of mental illness on receptive language (*p* = 0.0284 in the original study; *p* = 0.0292 using the *F* test as in this study, for comparison), suggesting that interactions between a variable potentially influencing the child's environment, which is also related to genetic risk of mental illness, and one domain may have an effect on another domain (Schiavon et al. 2024), but this may not be captured by testing for interaction with PGS for EA.

Overall, our study provides important insight into the roles of modifiable and non‐modifiable factors in child development. We show that the quality of the home environment measured by HOME (modifiable) has a much stronger association with developmental outcomes than genetic predisposition to educational attainment (non‐modifiable), and that the former is associated with a larger proportion of the variance in developmental domains across children. It is important to note that some genetic factors may be influencing the HOME score itself; however, this does not change the conclusions of our study, as the effect on the child is still environmental, unless the same genetic factors that influence HOME should also influence language, motor function, cognition, and social behavior. If such genetic factors do exist, they are more likely to be related to risk of schizophrenia and bipolar disorder in some of the families; however, this should be controlled for by the inclusion of the familial high‐risk status in our models, which further suggests that the observed effects cannot be explained only by the caregivers' mental health. Our findings are in line with recent literature indicating a major influence of modifiable environmental factors on neurodevelopmental outcomes, which may be mediated by a range of interdependent biological processes such as the over‐activation of the hypothalamus‐pituitary–adrenal axis, effects of the microbiome on the gut–brain axis, neuroplasticity, and epigenetic changes (Christensen et al. 2024).

### 4.1 Strengths and Limitations

Our study is, to our knowledge, the first study to examine the four investigated developmental domains simultaneously as captured by PCA, while modelling measures of both environmental and genetic factors together (as well as a possible interaction between them) as predictors, particularly in the context of familial risk of severe mental illness. As such, it provides important insights into the interplay between these factors in early development. Our results highlight the importance of supporting families in providing children with an appropriate home environment and interaction with caregiving figures, as this may improve neurodevelopmental outcomes. Some limitations should be noted as well. Firstly, our study is affected by some limitations inherent to the tests we used, for example, TROG‐2 being a test of receptive language only, and the RIST being a screening test. With regards to the results for the genetic predictor, the association between multiple developmental domains and PGS for EA was generally weaker than the associations with HOME, and the former was associated with considerably less variance across the children. This is likely influenced by the sample size, as well as the genetic architecture of EA itself; as most PGSs do, the PGS for EA includes some noise in addition to the true genetic signal, and it was not expected to fully capture all the genetic factors underlying the four domains, but rather only those factors that would overlap with genetic factors influencing EA. It has also been shown that the GWAS for EA based on which we computed the PGS captured more than direct genetic effects for EA, and that the associations in that GWAS were inflated partly due to assortative mating, and possibly due to the rearing environment (Lee et al. 2018). That said, some of these biases could still have a genetic origin (albeit one indirectly affecting EA in the offspring), and, for the purpose of using PGS as a prediction tool, including some of these non‐direct genetic effects might improve predictability (Abdellaoui et al. 2023).

## 5 Conclusions

Our study shows that considerable differences between children across multiple neurodevelopmental domains are influenced by differences in the home environment, measured using HOME, controlling for parental mental health, while a smaller part of the variance is associated with genetic factors associated with educational attainment. Our results suggest that children suffering from deficits in one or more developmental domains and their families should receive intervention and support in their domestic environment, which could potentially ameliorate the children's general development. This is especially important in light of the fact that developmental difficulties such as language and motor function disorders have been found to be associated with long‐term effects on the individual's mental health and functioning that can reach far into adulthood (Botting et al. 2016; Conti‐Ramsden et al. 2018; Mok et al. 2014; Tal‐Saban et al. 2014, 2018; Whitehouse et al. 2009). We thus hope that this study will encourage further investigations into the links between language, motor, cognitive, and social–behavioral functions, and the factors that may be influencing them, as well as interventional studies aimed at improving children's outcomes in these domains.

## Author Contributions

M.S. contributed to the study design, performed statistical analyses, analyzed the results, and wrote the paper; B.K.B., N.H., A.N.G., K.S.S., and D.E. contributed to the VIA 7 data collection and/or pilot study; T.W. designed and oversaw the genetic part of the VIA 7 study; K.J.P., A.A.E.T., J.R.M.J., and M.N. contributed to the conception of the VIA 7 project and its design, coordination, and funding applications; R.N. conceived and supervised the study, performed genetic quality control and imputation, statistical analyses, and polygenic score calculation, analyzed the results, and wrote the paper. All authors have read and approved the manuscript.

## Ethics Statement

The authors assert that all procedures contributing to this work comply with the ethical standards of the relevant national and institutional committees on human experimentation and with the Helsinki Declaration of 1975, as revised in 2008. The study was approved by the Danish Data Protection Agency and follows all laws concerning the processing of personal data. Permission to draw data from registers was granted by the Danish Ministry of Health. The study protocol was sent to the Danish Committee on Health Research Ethics, who decided that ethical approval was not needed due to the observational nature of the study. The genetic part of the study obtained ethical approval from the outset of the study and The Danish High Risk and Resilience Study–VIA 7 was later incorporated into the protocol (Arv og Miljø—genetics and environment) as an appendix, which has then been approved by the ethics committee (ARV OG MILJØ: betydning for psykisk sygdom hos børn og unge (H‐B‐2009‐026)). Written informed consent was obtained from all adult participants and from the legal guardians of participating children.

## Conflicts of Interest

The authors declare no conflicts of interest.

## Peer Review

The peer review history for this article is available at https://www.webofscience.com/api/gateway/wos/peer‐review/10.1111/ejn.70163.

## Supporting information


**Table S1** Results of the principal component analysis.
**Table S2** Information on PGS for EA and HOME total score for familial risk groups for mental illness.

## Data Availability

Access to the dataset used in the current study may be granted upon reasonable request to the principal investigators of the VIA project (https://viaundersøgelsen.org).
