# Supplementary material for: Environmental and Genetic Influences on Developmental Outcomes Across the Domains of Language, Cognition, Motor Function, and Social Behavior
Source: Eur J Neurosci. 2025 Jun 17;61(12):e70163. doi: 10.1111/ejn.70163 (PMC12172008; doi:10.1111/ejn.70163)
Supplement: Supplementary file 1 — Table S1 Results of the principal component analysis. Table S2 Information on PGS for EA and HOME total score for familial risk groups for mental illness. [file EJN-61-0-s001.pdf]

Supplementary material for: **Environmental and genetic influences on developmental outcomes across the domains of language, cognition, motor function, and social behavior**

- Note about the retaining principal components from the PCA for downstream analyses
- Supplementary Table S1: Results of the principal component analysis
- Supplementary Table S2: Information on PGS for EA and HOME total score for familial risk groups for mental illness

**Note about the retaining principal components from the PCA for downstream analyses**

To help decide which PCs to retain for downstream analyses, the *VSS* function of the R psych package v2.2.9 was used on the raw phenotype matrix after the imputation. This function employs many common methods for this purpose. It is important to note that these methods do not always agree with each other. The Very Simple Structure method (VSS) obtained a higher fit (0.82) with a complexity of 2 and two components than with a complexity of 1, where the best fit (0.71) was with one component, while with higher complexities, there was no visually discernable difference in the plot compared to the highest fit with a complexity of 2; additionally, a model with two components obtained the highest fit (0.82) for "factor fit of the complete model". In contrast, Velicer's minimum average partial (MAP) method had a minimum value of 0.13 with one component, and a minimum value for the Bayesian information criterion (BIC) (10.87) was achieved with one component. No other method included in this analysis recommended retaining three or four components. Based on these analyses and guidelines, and because our analyses of the PCs used them as independent outcomes rather than in one model together, we opted for an inclusive approach and used the first two PCs in downstream analyses, which together captured more than 70% of the variance.

**Supplementary Table S1: Results of the principal component analysis**

| | PC1 | PC2 | PC3 | PC4 |
|---|---|---|---|---|
| Language (TROG-2) | -0.5413914 | 0.4037400 | -0.1135247 | 0.72869849 |
| Motor function (MABC-2) | -0.4644198 | -0.5503097 | 0.6905971 | 0.06744767 |
| Cognition (RIST) | -0.5142837 | 0.5339653 | 0.1436705 | -0.65555481 |
| Social behavior (SRS-2) | 0.4761532 | 0.4990333 | 0.6996761 | 0.18627190 |
| Proportion of variance | 0.5257 | 0.2073 | 0.1537 | 0.1133 |
| Standard deviation (the square root of the eigenvalue) | 1.4501 | 0.9107 | 0.7841 | 0.6731 |

The values across the first four rows are the loadings of the PCs, i.e. the coefficients for the (standardized) original variables which can be used to calculate the PC scores, as in the output of the *prcomp* function in R.

**Supplementary Table S2: Information on EA PGS and HOME total score for familial risk groups for mental illness**

| Statistic | Schizophrenia | Bipolar disorder | Population-based controls |
|---|---|---|---|
| Standardized EA PGS – mean | -0.1947559 | 0.04933741 | 0.1463004 |
| Standardized EA PGS – standard deviation | 1.000397 | 1.016218 | 0.9669766 |
| HOME total score – mean | 45.13571 | 46.76344 | 49.12903 |
| HOME total score – standard deviation | 6.08715 | 4.6166 | 4.262049 |
